# Hygiene and Public Health

**Published:** 1889-12

**Authors:** 


*Hygiene and Public Health.* By LOUIS C. PARKES, M.D., D.P.H. Lond., Assistant Professor of Hygiene at University College. London: H. K. LEWIS. 1889.

In this volume of Lewis's practical series, Dr. Parkes has endeavoured "to occupy within a small space nearly the whole field of sanitary science, and to give the reader the opportunity of acquiring such an elementary knowledge on every topic as will enable him to refer with advantage to the larger and more abstruse text-books."

Starting with this object from the practical standpoint of a lecturer and examiner in his subject, Dr. Parkes has succeeded in writing a very clear and successful students' manual, in no sense of the word a "cram" book, and containing well-chosen references to larger works.

The chapters on Water, Disposal of Refuse, Air and Ventilation, and Communicable Diseases, are particularly good and up to date; while the practical examples worked out make a very useful feature, which might advantageously be extended.

The chapter on Statistics is confessedly incomplete, and all reference to the law of public health is perhaps wisely omitted, as these questions must be carefully studied in detail.

While, then, we must insist that it is impossible to do justice to so wide a subject in a small handbook, we must, on the other hand, admit that Dr. Parkes has well carried out the promise of his preface; and that the student could not do better at the outset of his public health studies than provide himself with this practical manual.

